# Detection and Genetic Characterization of Puumala Orthohantavirus S-Segment in Areas of France Non-Endemic for Nephropathia Epidemica

**DOI:** 10.3390/pathogens9090721

**Published:** 2020-09-01

**Authors:** Séverine Murri, Sarah Madrières, Caroline Tatard, Sylvain Piry, Laure Benoit, Anne Loiseau, Julien Pradel, Emmanuelle Artige, Philippe Audiot, Nicolas Leménager, Sandra Lacôte, Johann Vulin, Nathalie Charbonnel, Philippe Marianneau, Guillaume Castel

**Affiliations:** 1 ANSES—Laboratoire de Lyon, Unité Virologie, 69007 Lyon, France; severine.murri@anses.fr (S.M.); sarah.madrieres@anses.fr (S.M.); sandra.lacote@anses.fr (S.L.); Johann.VULIN@anses.fr (J.V.); Philippe.MARIANNEAU@anses.fr (P.M.); 2 CBGP, INRAE, CIRAD, IRD, Institut Agro, Université Montpellier, 34000 Montpellier, France; caroline.tatard@inrae.fr (C.T.); sylvain.piry@inrae.fr (S.P.); Laure.Benoit@Cirad.Fr (L.B.); anne.loiseau@inrae.fr (A.L.); julien.pradel@inrae.fr (J.P.); emmanuelle.artige@inrae.fr (E.A.); philippe.audiot@inrae.fr (P.A.); Nicolas.Lemenager@Cirad.Fr (N.L.); nathalie.charbonnel@inrae.fr (N.C.)

**Keywords:** *Myodes glareolus*, *Puumala* virus, diversity, molecular evolution, nephropathia epidemica, France

## Abstract

Puumala virus (PUUV) in Europe causes nephropathia epidemica (NE), a mild form of hemorrhagic fever with renal syndrome (HFRS). The incidence of NE is highly heterogeneous spatially, whereas the geographic distribution of the wild reservoir of PUUV, the bank vole, is essentially homogeneous. Our understanding of the processes driving this heterogeneity remains incomplete due to gaps in knowledge. Little is known about the current distribution and genetic variation of PUUV in the areas outside the well-identified zones of NE endemicity. We trapped bank voles in four forests in French regions in which NE is considered non-endemic, but sporadic NE cases have been reported recently. We tested bank voles for anti-PUUV IgG and characterized the S segment sequences of PUUV from seropositive animals. Phylogenetic analyses revealed specific amino-acid signatures and genetic differences between PUUV circulating in non-endemic and nearby NE-endemic areas. We also showed, in temporal surveys, that the amino-acid sequences of PUUV had undergone fewer recent changes in areas non-endemic for NE than in endemic areas. The evolutionary history of the current French PUUV clusters was investigated by phylogeographic approaches, and the results were considered in the context of the history of French forests. Our findings highlight the need to monitor the circulation and genetics of PUUV in a larger array of bank vole populations, to improve our understanding of the risk of NE.

## 1. Introduction

Over the last two decades, since the advent of reliable tools for orthohantavirus infection diagnosis, the human diseases associated with these zoonotic viruses have increasingly been recognized as a growing public health concern worldwide. In Europe, most human cases are caused by the Puumala virus (PUUV) [1]. This virus is the causal agent of nephropathia epidemica (NE) an attenuated form of hemorrhagic fever with renal syndrome (HFRS), thousands of cases of which are diagnosed in Europe annually [2]. PUUV possess a tri-segmented RNA genome of negative polarity (Figure S1): S (small) segment codes for the nucleocapsid protein and a small non-structural (NS)s protein [3]; M (medium) segment codes for a glycoprotein precursor (GPC) of the envelope glycoproteins Gn and Gc; and L (large) segment codes for the RNA-dependent RNA polymerase (RdRp) [4]. PUUV is transmitted to humans via aerosols contaminated with the excreta of its sole, specific natural reservoir, the bank vole, *Myodes glareolus*, a forest-dwelling rodent species [5]. Previous studies [6,7,8] have suggested that PUUV transmission is entirely horizontal in bank vole populations, through direct contact between individuals or indirect contamination via contact with infected feces or urine in the environment [7,9]. The relative importance of this indirect contamination seems to depend on local environmental conditions, which influence the survival of the virus outside its reservoir host [7]. In humans, the incidence of NE is highly heterogeneous at both the regional and continental scales [10,11], despite the spatially homogeneous distribution of *M. glareolus* throughout Europe [12]. In areas of PUUV endemicity, the virus circulates in both the reservoir and human populations. These areas cover most of northern Europe (Fennoscandinavia, the Baltic countries) and fragmented parts of Belgium, north-eastern France and western Germany.

The situation in France is particularly interesting, as the north-eastern quarter of the country lies within the western limits of the area of NE endemicity in Europe, but bank voles are found throughout the country with the exception of Mediterranean areas. A previous epidemiological study found that PUUV-infected rodents could be detected in non-endemic areas, with a high seroprevalence (i.e., areas with no reported cases of NE in humans, such as the forests of Sologne [13]). The reasons for this absence of NE cases remain unclear [14]. An understanding of the reasons for this discrepancy between PUUV circulation in bank vole populations and the occurrence of PUUV infections in humans would greatly facilitate public health management [15] and improve our ability to predict the risk of PUUV infection in humans [16,17].

The contemporary distribution of PUUV in French bank vole populations is probably underestimated, particularly in areas in which NE is currently considered non-endemic. Moreover, a geographic extension of the range of NE has recently been reported in France [18]. Cases of NE in humans have been reported outside the known endemic areas, in some parts of central France, for example, in the department of Loiret [19]. These findings suggest that the circulation of PUUV in bank vole populations is not sufficient for predicting the occurrence of NE human cases. Other factors, such as variations between genetic lineages of PUUV, may influence the human risk at local or regional scales [20]. The genetic features of the virus may also differentiate between areas of NE endemicity and non-endemicity in which PUUV circulates in the reservoir host populations. It has been shown that, even at small spatial scales, the persistence of PUUV variants over time may differ considerably between areas, with some populations displaying high rates of strain turnover, whereas others carry the same variants over many years [21,22,23]. In many viruses, particular variants seem to be associated with greater fitness, as assessed by their replication, excretion, and transmission rates, or their persistence over time in the host [24,25,26,27]. Viral variants may also differ in terms of their virulence in humans [28,29,30,31]. As such, their presence could constitute an indicator of the risk of virus emergence.

In this context, we investigated the distribution, genetic diversity and evolution of PUUV circulating in bank vole populations from six sites of three areas of France in which NE is non-endemic. The first two areas, corresponding to the north and south of the Morvan and Tronçais forests, respectively (see map), are located between known endemic (Jura) and non-endemic (Centre) areas, and have never been investigated. In these two areas, human cases with no obvious link to a known endemic area have recently been reported [19]. The other sites are located in the forests of Sologne (Loiret and Loir et Cher). PUUV has already been shown to circulate in bank vole populations from these areas [13], providing us with a unique opportunity to investigate the hypothesis that particular PUUV “sub-lineages” circulate in contexts of non-endemicity for NE. We also made use of the French PUUV sequence dataset updated in this study to investigate possible historical routes of PUUV dissemination in France. Phylogeographic reconstructions can help to clarify past dissemination events and to explain the current epidemiological profile of the virus [32,33]. This approach is, therefore, crucial, to improve our understanding of the evolutionary links between the different French PUUV “sub-lineages” associated with areas in which NE is endemic and non-endemic, and to assess the timescale over which they have appeared.

## 2. Results

### 2.1. Seroprevalence

Rodent trapping resulted in the capture of 254 bank voles at the six sites targeted in the three French forests located outside the zones of NE endemicity. In total, 30 animals were PUUV-seropositive (i.e., displayed a positive reaction to the recombinant PUUV N protein). The global seroprevalence was 11.8%, but seroprevalence was not homogeneous between sampling sites (Table 1). PUUV-seropositive bank voles were detected for the first time in the south of Morvan, and in two areas of forest in Sologne. All seropositive animals were tested in qRT-PCR and all were found positive. We found no “IgG+/qRTPCR−” or “IgG−/qRTPCR+” bank voles. 

No PUUV-seropositive bank voles were detected in the Tronçais, the north of Morvan (though only two bank voles were captured in this area) or in the northwestern part of the forest in Sologne.

### 2.2. Phylogenetic Analyses and Identification of Clade-Specific Amino-Acid Signatures

**Figure 1:**
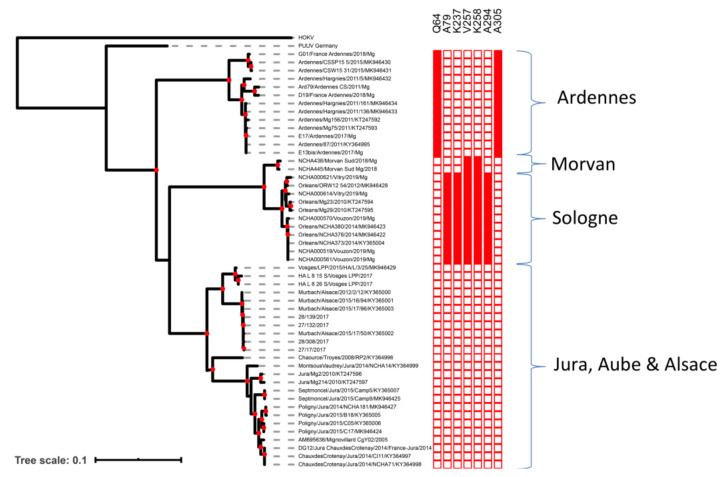
Phylogenetic tree of French PUUV S segment sequences (maximum likelihood (ML), general time reversible (GTR) + G model). Node supported by an approximate likelihood ratio test (aLRT) value > 0.75 are indicated with a red dot. Specific amino-acid signatures in the N proteins of each clade are reported on the heatmap.

Phylogenetic analyses of the French complete S coding sequences showed that the Ardennes clade was ancestral relative to the clades from other areas (Figure 1). Nucleoprotein sequences from this area contained the previously identified Q64 amino-acid signature [13,34,35] and another signature, A305, identified in this study.

Sequences from Morvan and Sologne were very similar and were included in the same genetic clade. Moreover, they shared two of the five amino-acid signatures (V257 and K258) specific to the Loiret sequences, based on the data obtained in previous studies [13] and here. The sequences from bank voles in southern Morvan were found to be very similar to the PUUV sequence isolated from a human patient from the northern part of the Morvan forest in 2012 (GenBank accession number MG923611). Estimated sequence identity reached 97.84% (nt) and 99.76% (aa), corresponding to a single amino-acid difference (position 264). The sequence obtained from the human patient also presented the V257 and K258 amino-acid signatures detected in bank voles from southern Morvan. Finally, as already reported [36], sequences from the endemic areas of the Jura, Aube and Alsace were genetically similar and clustered together in a third French cluster.

An analysis of evolutionary divergence for Vouzon (Sologne forest, NE-non-endemic area) between 2014 and 2019 showed no change in the amino-acid sequences of the PUUV S segments collected between these two years. The estimated divergence between the amino-acid sequences of PUUV collected in 2011 and 2018 in Hargnies (Ardennes forest, NE-endemic area) was 0.007 amino-acid substitutions per site (mean of 2.8 mutations in the N protein gene in seven years, 0.4 mutations per year).

### 2.3. Molecular Signatures of Selection 

No evidence of recombination was found in our dataset. We detected no positive selection signature for the N protein gene with most of the methods used (Adaptive Branch Site Random Effects Likelihood (aBSREL), Branch-site Unrestricted Statistical Test for Episodic Diversification (BUSTED), Fast Unbiased Approximate Bayesian Method (FUBAR), Fixed Effect Likelihood (FEL), Internal Fixed-Effects Likelihood (iFEL), Random Effects Likelihood (REL), Single Likelihood Ancestor Counting (SLAC)). A signature of positive selection on this gene was detected only with the Mixed Effects Model of Evolution (MEME) method, which can detect sites evolving under positive selection in a small proportion of the branches of the tree. MEME identified a single site that had evolved under positive selection in the branch corresponding to the Ardennes clade, at position 64. Conversely, a very large number of amino-acid sites in the N protein gene were found to display signatures of purifying selection (108 with the SLAC method to 186 with the REL method) (Table S2).

### 2.4. Phylogeography of PUUV at the National Scale in France

**Figure 2:**
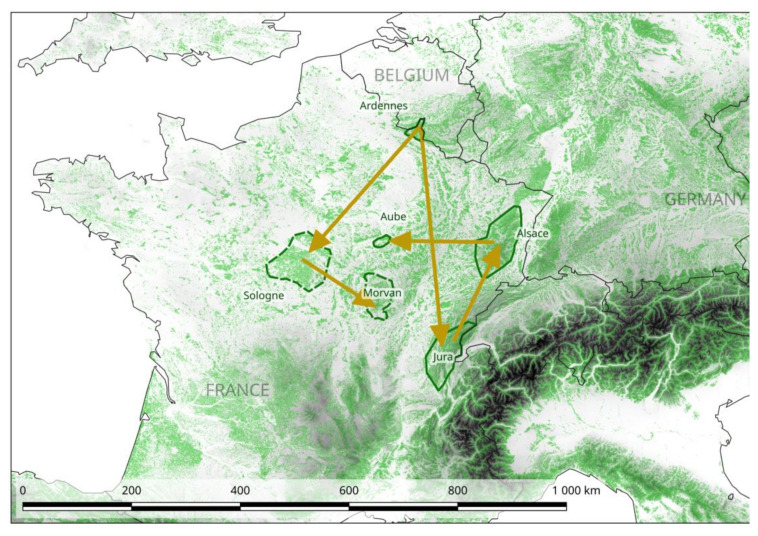
Phylogeography of PUUV in France. The scenario with highest support obtained with the Bayesian method (BEAST) program is represented on the map. Non-endemic areas for NE are surrounded by dotted lines (see Figure 3). This map was produced with Corine Land Cover (CLC) 2012, Version 2020_20u1 data, available from https://land.copernicus.eu/pan-european/corine-land-cover/clc-2012?tab=metadata [37].

The application of a Bayesian method (BEAST) to this enlarged dataset revealed that the Ardennes area was the best candidate area for the introduction of PUUV into France (Bayes factor (BF) = 3.1). We identified two potential routes of dissemination from this area. The first one involved spread through the areas of Sologne non-endemic for NE, and then through those of Morvan (BF = 2.5 and 10.2 respectively). The second expansion route involved spread through the large area in which NE is endemic, from Alsace and Aube to the Jura (Figure 2 and S2). However, inside this area, transitions between the different sites have similar probabilities and low BF, so it is difficult to retrace with confidence the steps of the route. In the scenario with the highest probability, the strains circulating in the Aube and Alsace areas originated from the Jura (BF for the Ardennes – Jura transition = 2.4 and for the Jura – Alsace transition = 4.8). 

The results obtained with the ML method (F81 algorithm) also supported this scenario (Figure S3). The time to most recent common ancestor (TMRCA) for the current French PUUV dated back to 598 years ago (95% highest posterior density (HPD) = 204 − 850). The clade including the Loiret and Morvan strains was found to be the most recent (98 years ago, 95% HPD = 42 − 194), whereas the clade including the Jura/Alsace/Aube was found to be of intermediate age (215 years ago, 95% HPD = 101 − 409). However, caution is required in the interpretation of these results, because the 95% HPD was large in each case.

The rate of PUUV evolution estimated over the entire tree was 2.57 × 10^−4^ substitution/site/year (s/s/y) (95% HPD: 1.04 × 10^−4^ – 4.16 × 10^−4^) under the relaxed clock model. The rates of evolution of the different clades were similar (2.56 × 10^−4^ s/s/y to 2.58 × 10^−4^ s/s/y). 

## 3. Discussion

In this study, we investigated whether PUUV circulates in bank vole populations from French geographic areas in which the presence of this virus had never been tested, but in which its circulation was considered likely, due to proximity to areas of NE endemicity or the detection of recent human cases in nearby areas. We provide the first evidence of PUUV circulation in bank vole populations from one French area currently considered to be non-endemic for NE: the southern Morvan forest. We also confirmed PUUV circulation in various parts of the Sologne forest, another region of France in which NE is considered non-endemic, but in which a high seroprevalence for PUUV in bank vole populations had already been reported [13]. These results show that PUUV circulates in areas in which NE is not endemic in France, highlighting the importance of not limiting studies of the distribution of PUUV in France (and probably in Europe) to areas in which NE is known to be endemic. 

These results also raise questions about the almost total absence of human cases of NE recorded in these two French areas, despite the circulation of PUUV in its reservoir host at prevalence similar to that in the areas of France in which NE is endemic [13]. One possible reason for this discrepancy may be that human cases are being missed because local doctors do not know enough about NE. Several serological studies have already suggested that large numbers of undiagnosed human Hantavirus infections occur in various countries [38,39,40,41,42]. However, we think that a significant number of serious cases of NE would undoubtedly have led to medical investigations and more accurate diagnosis, so this possibility seems unlikely. There are, therefore, probably factors limiting the number of NE cases in these areas in which NE is non-endemic, by decreasing PUUV transmission from bank voles to humans and/or by decreasing PUUV virulence in infected humans.

Differences in human susceptibility to PUUV infections exist (see [43] for a review) and might account for the absence of NE cases despite the circulation of PUUV. However, such immunological heterogeneity between human populations is highly unlikely at the geographic scale of this study. Besides, experimental studies have provided evidence to suggest that bank voles from areas in which NE is non-endemic may be less susceptible to PUUV infection [44,45], resulting in lower levels of PUUV excretion by these bank voles, decreasing both PUUV persistence in bank vole populations and transmission to humans. This hypothesis should now be investigated in bank voles from the Sologne and Morvan forests in France.

The hypothesis that genetically differentiated PUUV strains may display different levels of transmission from bank voles or virulence in humans has been little explored. Different viral strains may have different dynamics or be excreted to different extents by their hosts [46]. The absence of laboratory strains closely resembling wild strains would make studies of this possibility difficult for PUUV, but further investigations are nevertheless required. Horling et al. [47] suggested that there is unlikely to be any “human PUUV genetic specificity”, as no genetic difference was found between the sequences of PUUV recovered from humans, and those from rodents in the same area. However, more recently, Klempa et al. [48] showed that two genetic lineages of Dobrava-Belgrade orthohantavirus (DOBV) could display very different levels of virulence in humans. Our findings confirm those of previous studies highlighting the circulation of several highly differentiated clusters of PUUV in different areas of France. We also showed that the PUUV strains circulating in the Morvan forest had nucleoprotein sequences phylogenetically close to those detected in the Sologne forest, with amino-acid signatures in common that were specific to these areas in which NE is non-endemic. These genetic features, which differentiate between PUUV from areas in which NE is endemic and those in which it is non-endemic, might result in differences in the virulence of these strains in humans. Like other HFRS, NE is a hyperinflammatory syndrome, with excessive immune activation, including massive cytokine responses (cytokine storm) and the activation of cytotoxic lymphocytes [49,50,51]. The PUUV N protein is highly immunogenic in both bank voles and humans, eliciting a strong humoral response in infected patients [52,53,54,55], but it also plays an important role in the cell-mediated immune response [56,57]. We currently have no firm evidence that the amino acids involved in the signatures specific to areas of NE endemicity/non-endemicity affect the virulence of the corresponding genotypes, but some are located in the major antigenic domain or in the hypervariable domain of the N protein, areas of the protein known to contain both important T-cell and B-cell epitopes [52,53,55,56,57]. As such, these amino acids could interact with the human immune system and mediate differences in virulence. Further investigations are required to explore this hypothesis and, more broadly, to link PUUV genomic variations (for the N protein sequence, but also for the sequences of the less studied glycoproteins and polymerase) with potential functional consequences for both bank vole/PUUV interactions and virulence in human infections.

Another intriguing issue relating to PUUV circulation in these areas of France non-endemic for NE was the absence of temporal genetic variability over a five-year period in the Sologne forest (Vouzon). According to the estimated rate of nucleotide substitution in PUUV, we expected to detect S-segment changes between 2014 and 2019 in samples of the same bank vole population [4,22]. We observed no significant difference in long-term substitution rates (nucleotides) between the different French clades, but, interestingly, the PUUV genotypes surveyed in an area non-endemic for NE remained similar, with no mutations affecting the amino-acid sequence detected in five years, whereas a mean of 0.4 mutations per year was recorded between 2011 and 2018 at a site (Hargnies) sampled in the French Ardennes area in which NE is endemic. A similar pattern of low/no temporal variability has already been described over a similar period in Northern Sweden [21]. The low level of genetic variability of PUUV may be driven by epidemiological features related to bank vole population dynamics [21]. If confirmed, this genetic stability of PUUV over time may have important implications for NE epidemiology. Assuming that the bank voles in areas non-endemic for NE carry PUUV genotypes that are less virulent or less frequently transmitted to humans, this high stability would also result in the maintenance of small numbers of NE cases. All these hypotheses require confirmation through studies on a larger number of sampling sites and long-term surveys. It is also important to bear in mind that bank voles and PUUV have been co-evolving for thousands of years, whereas humans are only accidental hosts without human-to-human transmission. There is, therefore, no adaptation as yet in humans, and following the host jump the virus is likely to be poorly adapted to its accidental human host [58]. Consequently, any mutations acquired by PUUV during its evolutionary history that would have led to a change in virulence in humans [59] must therefore be the result of chance, rather than selection.

PUUV has long been known to exist as a swarm of closely related mutants (quasi-species) in any given host [4,60,61], but the role of PUUV quasi-species in virulence has yet to be explored. As in other viruses [61], the intra-host genetic diversity of PUUV may, at least partly, govern the virulence of the virus in humans, by interfering with the host immune system via antigenic variation [51], or, more generally, by favoring adaptation to a new host [62]. Most studies exploring PUUV diversity to date have been based on Sanger sequencing and were therefore unable to assess this intra-host genetic diversity. The advent of high-throughput sequencing is opening up promising new opportunities for improving investigations of the impact of PUUV intra-host diversity on the transmission of this virus between bank voles, and between bank voles and humans, and on its virulence in humans.

Local abiotic factors (e.g., temperature, moisture, protection from UV light) may also affect the epidemiological characteristics of PUUV and the number of NE cases, and of PUUV transmission in particular, by influencing bank vole abundance and viral persistence outside the host [11,63,64,65]. Ecological niche modeling has been used to predict the distribution of NE cases in Europe [66,67]. In France, these models have predicted the largest numbers of NE cases for the northeastern part of the country, but also a few cases in some unexpected areas (southern and central France). These unexpected cases were interpreted as corresponding to suitable areas in which NE may already occur without having been noticed, or in which all the necessary conditions for NE emergence are met [66]. The Morvan forest is located close to one of these areas predicted to be ‘at risk’, but the Sologne forest was not. This finding suggests that environmental factors may partly explain the risks of infectious disease emergence, but that the whole pathosystem, including reservoir population dynamics, evolution, host-pathogen interactions, and sociological features, must be considered if we are to develop a profound understanding of these risks. For example, several studies have shown that genetically differentiated lineages may have different environmental preferences, suggesting that phylogeographic information should be taken into account [68,69] to improve the prediction of areas at risk of disease emergence, under the hypotheses of virus diffusion and climate change [69,70,71,72].

Another aim of this study was to deepen our understanding of the evolutionary history of these French PUUV clusters associated with different epidemiological situations for NE. The phylogeographic approach provided new insight into the origin and epidemic spread of PUUV. These results can provide the basis for predicting changes in NE epidemiology in France on the basis of the contemporary history of PUUV [73]. Castel et al. [74] previously showed that PUUV probably arrived in France hundreds of years ago, following postglacial recolonization by bank voles from the Central Europe refugia [74]. We show here that the Ardennes forest is the most likely origin of the dissemination of PUUV in France. From the Ardennes, we identified two potential routes of dissemination in France, one leading to two areas in which NE is non-endemic, in the Sologne and Morvan forests, and the second one leading to areas of NE endemicity in the Jura, Alsace and Aube. This finding is consistent with a recent phylogenetic study from Germany that also suggested that local German PUUV strains had undergone long-term parapatric evolution rather than a rapid expansion in Germany [75].

This history is strongly linked to the forests in which the bank voles settled and in which PUUV evolved. We found that the ancestors of the PUUV strains currently circulating in Morvan and Sologne (non-endemic for NE) were more recent than those of the PUUV strains circulating in the Jura and Ardennes (endemic for NE). This result should be interpreted with caution, however, as the estimated 95% HPD values associating with the dating results were large. Indeed, methods for estimating mutation rates from the tips of the tree (leaves) are known to be less accurate for estimating ancient divergence events in phylogenies, potentially leading to a considerable underestimation of divergence for viral ancestors [76,77,78,79]. Moreover, the strong purifying selection acting during the evolution of French PUUV S-segments that we detected, as already reported for PUUV and other hantaviruses [23,26,77], may complicate molecular dating [77,78]. However, it is interesting to relate these estimates to the history of French forests. The area under forest has greatly changed in France, at least since the time of the Roman Empire, with significant decreases and increases due to human activities [80,81]. The biodiversity of current forests may still be influenced by factors relating to ancient cultivation almost two millennia ago [80,82]. We found that the timing of the genetic differentiation of the current French PUUV clusters (between 600 and 100 years ago) corresponded roughly to a period during which the forests started to grow again after era long period of deforestation (the 1669 ordinance of Louis XIV concerning “waters and forests” which led to a revival of French forests). The Morvan and Sologne forests, in particular, contain a large proportion of recent plots, due to extensive reforestation since the middle of the 20th century and the 18th century, respectively [81], potentially explaining their more recent PUUV ancestor.

In conclusion, PUUV is currently circulating in areas of France in which NE is non-endemic, and the local PUUV strains from these areas display both specific and shared amino-acid signatures. In one of the surveyed areas non-endemic for NE, these genotypes seemed to have been strongly conserved over the last five years. The local distribution of French clades may reflect contrasting changes in French forests since the introduction of a common ancestor, leading to the local evolution of each cluster for hundreds of years. We recommend the prospection and monitoring of the circulation and genetic evolution of PUUV over wide geographic areas in French and European rodent populations. These surveys should improve our knowledge of NE epidemiology and help to improve the diagnosis, prevention and cure of cases of NE in areas in which NE is currently considered to be non-endemic.

## 4. Materials and Methods

### 4.1. Ethics Statement

All animal studies were conducted in accordance with French and European regulations on the care and protection of laboratory animals (French Law 2013-118 from 1 February 2013 and Directive 2010/63/EU from 22 September 2010). All the animal procedures were performed with the approval of the competent ethics committee (Languedoc-Roussillon) and the “Direction des Services Vétérinaires de l’Hérault” (D 34-169-1 Agreement).

### 4.2. Rodent Sampling

**Figure 3:**
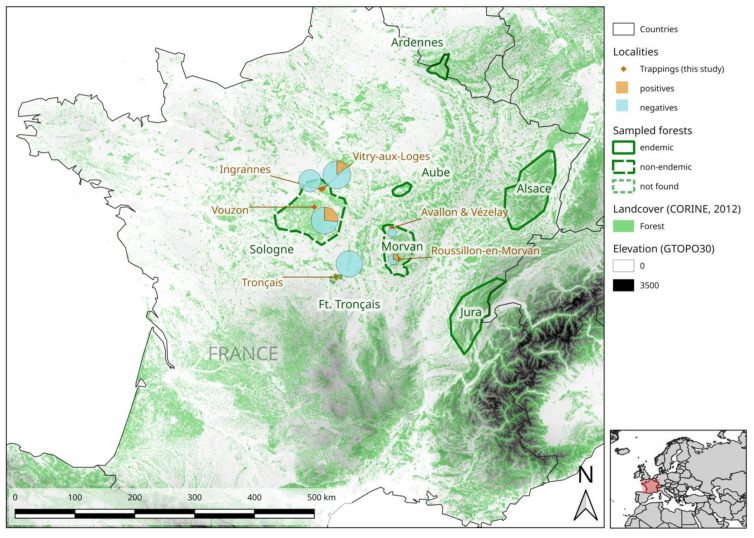
Map of France showing the nephropathia epidemica- (NE)-endemic and non-endemic areas studied. The sampling sites for this study are represented by red diamonds. PUUV seroprevalence at each sampling site is represented by a pie chart, the area of which is proportional to the number of captures. The precise GPS coordinates of each site are shown in Table 1. This map was produced with Corine Land Cover (CLC) 2012, Version 2020_20u1 data, available from https://land.copernicus.eu/pan-european/corine-land-cover/clc-2012?tab=metadata [37].

Bank voles were trapped in September and October 2018 and 2019, at six sites located in three forests within three areas in which NE is considered non-endemic (Figure 3, Table 1), but sporadic cases of NE have recently been reported [19,35].

We set up 10 or 11 lines of 20 French Agricultural Research Institute (INRA) live traps, fitted with dormitory boxes and baited with apple and sunflower seeds, separated by an interval of about 5 m. The traps were checked each morning. The trapped voles were anesthetized, and blood samples were collected by retro-orbital sinus puncture for further serological analyses. Whole blood was stored at 4 °C until centrifugation for serum isolation. The voles were then killed by cervical dislocation. Their sex was determined, they were weighed and their lungs were removed. Serum samples and lungs were immediately placed on dry ice for transport, and were subsequently stored at −80 °C for further serological and virological analyses.

### 4.3. Serological and Virological Analyses

Serum samples were diluted to 1/100 and screened with IgG ELISA, as described by [83]. The plates were coated with PUUV recombinant nucleocapsid protein [44]. A negative control was also included. Samples were considered positive if optical density was greater than 0.1. The lungs are target organs for PUUV replication [6,44,84]. We therefore extracted viral RNA from the lungs of seropositive voles, with the QIAamp Viral Mini Kit (Qiagen). Quantitative RT-PCR (RT-qPCR) was performed with 2.5 µL viral RNA and the SuperScript III One-Step RT-PCR system with the Platinum High Fidelity Taq polymerase (Invitrogen), on a LightCycler 480 machine. The S segment was amplified from samples testing positive by RT-qPCR with low CT values (<25), only one sample from Morvan and 8 samples from Vitry and Vouzon). So one or two samples of each country were amplified, by RT-PCR with the Titan One-Tube RT-PCR Kit and nested PCR with Taq polymerase. Amplicons were sequenced by the Sanger method. The primers and cycling conditions used for sequencing are described in [36], with a modification for fragment two (Primer Puu1F12-Or: CAAGGGAACTCGTATTCGA; Puu1R5-Or: CATCACCCAAATGAAAGTGATCT).

The complete S coding sequences of the samples have been deposited in the GenBank database under accession numbers MT742561-MT742577 (Table S1).

### 4.4. Phylogenetic Analyses and Detection of Clade-Specific Amino-Acid Signatures

We performed phylogenetic analyses on the dataset composed of all the complete S segment sequences from PUUV (open reading frame of 1299 nt encoding the viral nucleocapsid protein) available from GenBank and corresponding to French bank voles, and the new S segment sequences obtained in this study (Figure S1). The 50 selected sequences, their sampling date and geographic origin are shown in Table S1. Sequences of Asian (AB010731) and German (EU439968) PUUV strains were used as outgroups in all analyses.

A multiple sequence alignment was generated with the CLUSTAL Omega alignment program implemented in SEAVIEW v4.6.1 [85]. Nucleotide sequences were translated into amino-acid sequences and analyzed with SEAVIEW v4.6.1. Phylogenetic reconstruction was performed by a maximum likelihood (ML) approach in PHYML v3.0 [86], with a statistical approximate likelihood ratio test (aLRT) for branch support. The optimal substitution model was identified as the general time reversible (GTR) + G model with the SMS software [87]. We applied rate heterogeneity, using a discrete gamma distribution with four rate categories, and an estimated gamma shape parameter alpha of 0.135. Nucleotide frequencies were optimized from the dataset. 

Phylogenetic trees were visualized with FIGTREE v1.4.3 [88].

We looked for amino-acid signatures specific to particular French clades with VESPA (Viral Epidemiology Signature Pattern Analysis) [89].

A heatmap representing the signatures identified for each sequence of the tree was then created with EVOLVIEW v.2 [90].

### 4.5. Detection of Signatures of Selection

We investigated whether French PUUV strains had evolved under lineage-specific positive (diversifying) selection, or whether particular sites in the gene encoding the N protein had experienced positive selection in only one or a subset of lineages [91]. We searched for molecular signatures of selection potentially associated with different dissemination routes, by several methods available from the DATAMONKEY webserver [92,93]. Recombination events was first search using the GARD method [94]. We used: (i) several codon-based maximum-likelihood methods inferring sites under either positive or negative selection: the single likelihood ancestor counting (SLAC), fixed effect likelihood (FEL), random effects likelihood (REL) and internal fixed-effects likelihood (IFEL) methods. This last method is similar to FEL, except that selection is tested only along internal branches of the phylogeny. We also used the mixed effects model of evolution (MEME). This branch-site model is an extension of FEL in which a proportion of the branches at a site are allowed to evolve neutrally or under negative selection, whereas the others can evolve under diversifying selection. It can therefore detect signatures of episodic selection, even when most of the lineages are subject to purifying selection; (ii) A codon-based Bayesian approach: the fast unbiased approximate Bayesian (FUBAR) method, a very fast approximate hierarchical Bayesian method based on a Markov chain-Monte Carlo (MCMC) routine that ensures robustness against model misspecification by averaging over a large number of predefined site classes; (iii) a “branch-site” model: the adaptive Branch-Site Random Effects Likelihood (aBSREL); and (iv) A “gene-wide” model: the branch-site unrestricted statistical test for episodic diversification (BUSTED).

### 4.6. Estimation of Evolutionary Divergence

We included PUUV sequences previously obtained from bank voles sampled in Vouzon (NE non-endemic area) and Hargnies (NE endemic area) (sampled for another unpublished study based on the same sampling and sequencing protocol) to analyze the evolutionary divergence of PUUV sequences between 2011 and 2019.

Estimates of evolutionary divergence were calculated with a function implemented in MEGAX [95]. The number of amino-acid substitutions per site was calculated by averaging over all sequence pairs between groups, with a Poisson correction model. All positions containing gaps and missing data were eliminated. All other parameters were set to their default values.

It was difficult to assign human sequences to a particular geographic site. We therefore chose to add only PUUV sequences from our dataset obtained from the bank voles trapped in this study. However, we also used MEGAX to analyze the divergence of PUUV sequences isolated from bank voles in Morvan (MT742572-MT742573) and PUUV sequences isolated from a human patient in living in the northern part of Morvan in 2012 (GenBank number MG923611).

### 4.7. Phylogeographic Analyses

We analyzed PUUV dissemination scenarios in areas of France endemic and non-endemic for NE, using the dataset of sequences encoding the complete S segment, covering all known areas in which NE is known to be endemic and the areas currently considered to be non-endemic in which the virus is circulating. Temporal signal for the dataset was first checked using the TEMPEST v.1.5.3 program [96]. Bayesian phylogeographic reconstructions were then performed with the Metropolis-coupled Markov Chain-Monte Carlo (MCMC) method in BEAST package v1.10.4. [97]. BEAUTi v1.10.4 [97] was used to define parameter settings and to generate BEAST input files. The dataset was analyzed under the GTR +G model and a lognormal relaxed clock (allowing branch lengths to vary according to an uncorrelated lognormal distribution). The performance of several combinations of tree priors and parameters was assessed with path sampling (PS) and stepping-stone sampling (SS) marginal likelihood estimators [98]. Reconstruction was performed with the non-parametric and highly flexible coalescent Bayesian skyline tree prior [99], which allows stochastic variation of the size of the population over time, together with an asymmetric diffusion model in which the transition rates between locations are not reversible. The spatial information for PUUV was thus used to infer geographic patterns of PUUV dispersal, by fitting a standard continuous-time Markov chain (CTMC) model. We used a Bayesian stochastic search variable selection (BSSVS) procedure to assess the significance of each migration route in a Bayes factor (BF) test [100]. The dates on which the sequenced PUUV were sampled were used to estimate the rate of evolution and the ancestral time corresponding to the internal nodes. A random tree was used as the starting tree. In the absence of appropriate information, the prior location.clock.rate was set to default values (CTMC rate reference). All other priors were set at default settings. We performed five independent runs of 30 million generations, with parameters sampled every 5000 generations, to increase effective sample size (ESS) to 200. Parameters and convergence were evaluated with TRACER v1.7.1. [101] Summary maximum clade credibility (MCC) trees were generated with TREEANNOTATOR v1.10.4 [97] after discarding the first 10% of the trees as burn-in, as determined graphically with TRACER v1.7.1 and a combination of the three runs by LOGCOMBINER v1.10.4. [97]. We used SPREAD3 [102] to calculate BFs and posterior probabilities (PPs) from BSSVS results, to identify statistically significant epidemiological links between separate locations. The rates of evolution for the entire tree and for each separate clade were estimated with TREESTATS v1.10.4 [97].

We also performed phylogeographic reconstruction by maximum likelihood methods (F81-like model of character evolution), with the recently published PASTVIEW program [103], for comparison of the results obtained with those generated by the Bayesian method described above. Analyses were performed on the rooted phylogenetic tree previously computed by PHYML, with annotated tips (geographic origin of each isolate). PASTVIEW results were visualized and compared with results obtained with other methods, to find common patterns in multiple evolutionary scenarios with the dedicated functions of the software.

## Figures and Tables

**Table 1:** Puumala virus (PUUV) seroprevalence at each sampling site. Number of seropositive bank voles that are also positives in qRT-PCR is indicated in parentheses.

Sampling Site	Geographic Coordinates	Forest	Sampling Period	Number Seropositive for PUUV/Number of Bank Voles Trapped	Number of Seropositive Bank Voles That Are Positive in qRT-PCR
Roussillon-en-Morvan	47.01° N, 4.07° E	Morvan (South)	September 2018	3/10	3/3
Avallon/Vézelay	47.47° N, 3.86° E	Morvan (North)	September 2018	0/2	-
Tronçais	46.66° N, 2.74° E	Tronçais	September 2019	0/62	-
Vouzon	47.66° N, 2.10° E	Sologne (South)	October 2019	17/65	17/17
Vitry aux Loges	47.96° N, 2.26° E	Sologne (North-East)	October 2019	10/70	10/10
Ingrannes	47.94° N, 2.21° E	Sologne (North-West)	October 2018	0/45	-

## Supplementary Materials

The following are available online at https://www.mdpi.com/2076-0817/9/9/721/s1, Figure S1: Genomic organization of PUUV, Figure S2: Bayesian MCC time-scaled discrete phylogeographic tree for French PUUV S-segment sequences. The phylogeny branches are colored according to the location of their descendant nodes and bars on the nodes represent the 95%HPD. The legend indicates the regions to which the colors correspond, Figure S3: Tree-like representation of transitions based on the ancestral geographic reconstructions obtained with ML methods, and F81 algorithms, (PastView program). Pie-charts with a percentage display the proportion of subsequent strains annotated with the same ancestral annotation, Table S1: List of the N coding sequences (S segment) of French PUUV used for phylogenetic analyses and ancestral reconstructions. Sampling dates and areas are indicated. The S segments sequenced for this study are indicated by an asterisk (*), Table S2: Sites under positive and negative selection, for the French PUUV strain dataset (N protein, 433 aa). SLAC: single-likelihood ancestor counting; FEL: fixed-effects likelihood; MEME: mixed effects model of evolution; FUBAR: fast unbiased approximate Bayesian; aBSREL: adaptive branch-site random effects likelihood; BUSTED: branch-site unrestricted statistical test for episodic diversification.
