# Supplementary material for: Detection and Genetic Characterization of Puumala Orthohantavirus S-Segment in Areas of France Non-Endemic for Nephropathia Epidemica
Source: Pathogens. 2020 Sep 1;9(9):721. doi: 10.3390/pathogens9090721 (PMC7559001; doi:10.3390/pathogens9090721)
Supplement: Supplementary file 1 [file pathogens-09-00721-s001.zip › Supplementary/Table S1.docx]

| **Isolate** | **Genbank Number** | **Sampling year** | **Region** |
| --- | --- | --- | --- |
| Murbach/Alsace/2012/2/12 | KY365000 | 2012 | Alsace |
| 27/17/2017 | MT742561* | 2017 |  |
| 28/139/2017 | MT742563* | 2017 |  |
| 28/308/2017 | MT742564* | 2017 |  |
| 27/132/2017 | MT742562* | 2017 |  |
| Murbach/Alsace/2015/16/94 | KY365001 | 2015 |  |
| Murbach/Alsace/2015/17/50 | KY365002 | 2015 |  |
| Murbach/Alsace/2015/17/96/KY365003 | KY365003 | 2015 |  |
| Vosges/LPP/2015/HA/L/3/25 | MK946429 | 2015 |  |
| HA_L_8_15_S/Vosges_LPP/2017 | MT742565* | 2017 |  |
| HA_L_8_23_S/Vosges_LPP/2017 | MT742566* | 2017 |  |
| Ardennes/87/2011 | KY364995 | 2011 | Ardennes |
| Ardennes/Mg156/2011 | KT247592 | 2011 |  |
| Ardennes/Mg75/2011 | KT247593 | 2011 |  |
| Ardennes/Hargnies/2011/136 | MK946433 | 2011 |  |
| Ardennes/Hargnies/2011/161 | MK946434 | 2011 |  |
| Ardennes/Hargnies/2011/5 | MK946432 | 2011 |  |
| Ardennes/CSSP15_5/2015 | MK946430 | 2015 |  |
| Ardennes/CSW15_31/2015 | MK946431 | 2015 |  |
| E13bis/Ardennes/2017/Mg | MT742574* | 2017 |  |
| E17/Ardennes/2017/Mg | MT742575* | 2017 |  |
| D19/France_Ardennes/2018/Mg | MT742576* | 2018 |  |
| G01/France_Ardennes/2018/Mg | MT742577* | 2018 |  |
| Chaource/Troyes/2008/RP2 | KY364996 | 2008 | Aube |
| Mignovillard_CgY02/2005 | AM695638 | 2005 | Jura |
| Jura/Mg2/2010 | KT247596 | 2010 |  |
| Jura/Mg214/2010 | KT247597 | 2010 |  |
| ChauxdesCrotenay/Jura/2014/CI11 | KY364997 | 2014 |  |
| ChauxdesCrotenay/Jura/2014/NCHA71 | KY364998 | 2014 |  |
| DG12/Jura_ChauxdesCrotenay/2014/France-Jura/2014 | MK946426 | 2014 |  |
| MontsousVaudrey/Jura/2014/NCHA14 | KY364999 | 2014 |  |
| Poligny/Jura/2014/NCHA181 | MK946427 | 2014 |  |
| Poligny/Jura/2015/B18 | KY365005 | 2015 |  |
| Poligny/Jura/2015/C05 | KY365006 | 2015 |  |
| Poligny/Jura/2015/C17 | MK946424 | 2015 |  |
| Septmoncel/Jura/2015/Camp5 | KY365007 | 2015 |  |
| Septmoncel/Jura/2015/Camp9 | MK946425 | 2015 |  |
| Orleans/Mg23/2010 | KT247594 | 2010 | Loiret |
| Orleans/Mg29/2010 | KT247595 | 2010 |  |
| Orleans/ORW12_54/2012 | MK946428 | 2012 |  |
| Orleans/NCHA373/2014 | KY365004 | 2014 |  |
| Orleans/NCHA376/2014 | MK946422 | 2014 |  |
| Orleans/NCHA380/2014 | MK946423 | 2014 |  |
| NCHA000519/Vouzon/2019/Mg | MT742567* | 2019 |  |
| NCHA000561/Vouzon/2019/Mg | MT742568* | 2019 |  |
| NCHA000570/Vouzon/2019/Mg | MT742569* | 2019 |  |
| NCHA000614/Vitry/2019/Mg | MT742570* | 2019 |  |
| NCHA000621/Vitry/2019/Mg | MT742571* | 2019 |  |
| NCHA438/Morvan_Sud/2018/Mg | MT742572* | 2018 | Morvan |
| NCHA445/Morvan_Sud_Mg/2018 | MT742573* | 2018 |  |

Table S1. List of the N coding sequences (S segment) of French PUUV isolates used for phylogenetic analyses and ancestral reconstructions. Sampling dates and areas are indicated. The isolates sequenced for this study are indicated by an asterisk (*).
