# Supplementary figures and images for: Detection and Genetic Characterization of Puumala Orthohantavirus S-Segment in Areas of France Non-Endemic for Nephropathia Epidemica
Source: Pathogens. 2020 Sep 1;9(9):721. doi: 10.3390/pathogens9090721 (PMC7559001; doi:10.3390/pathogens9090721)

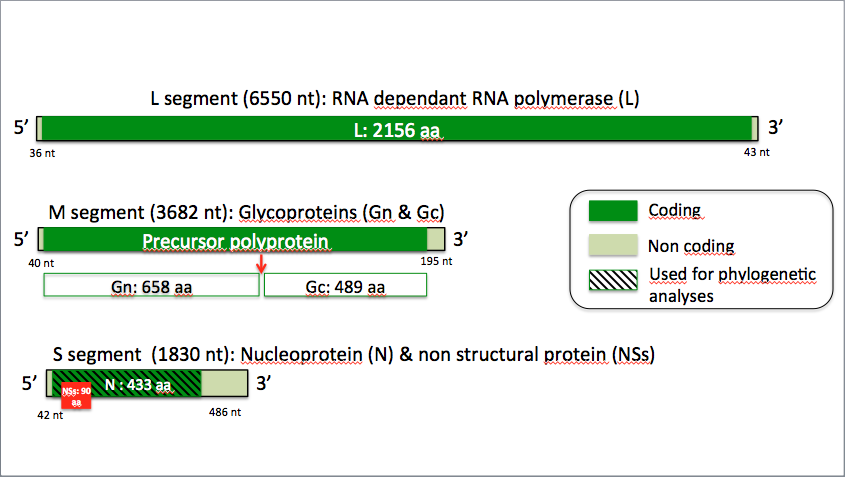

Supplement: Supplementary file 1 [file pathogens-09-00721-s001.zip › Supplementary/Figure S1.png]

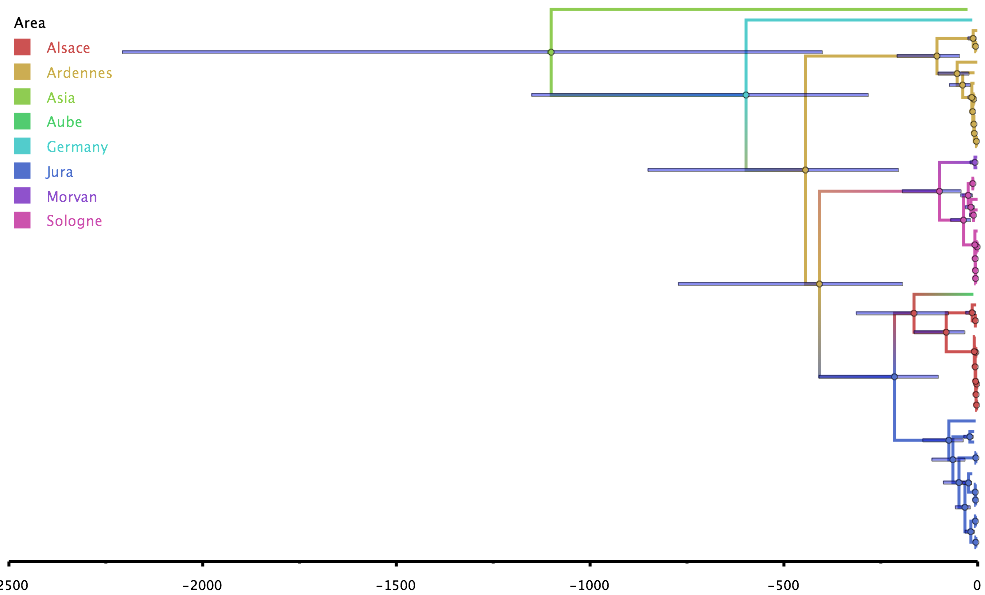

Supplement: Supplementary file 1 [file pathogens-09-00721-s001.zip › Supplementary/Figure S2.png]

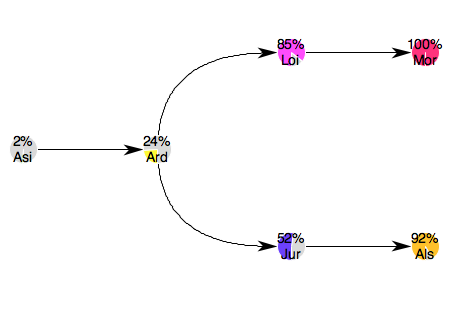

Supplement: Supplementary file 1 [file pathogens-09-00721-s001.zip › Supplementary/Figure S3.png]
