# Supplementary material for: Detection and Genetic Characterization of Puumala Orthohantavirus S-Segment in Areas of France Non-Endemic for Nephropathia Epidemica
Source: Pathogens. 2020 Sep 1;9(9):721. doi: 10.3390/pathogens9090721 (PMC7559001; doi:10.3390/pathogens9090721)
Supplement: Supplementary file 1 [file pathogens-09-00721-s001.zip › Supplementary/Table S2.doc]

| **Evolutionary test** | **No. of codons under** **negative/purifying selection** | **No. of codons under**  **positive/diversifying selection** |  |  |
| --- | --- | --- | --- | --- |
| **FEL** | 222* | 0* |  |  |
| **SLAC** | 108* | 0* |  |  |
| **FUBAR** | 272** | 0** |  |  |
| **aBSREL** | NA | 0***  (72 branches tested) |  |  |
| **BUSTED** | NA | 0 (LRT, p-value = 0.500 ≥ .05) |  |  |
| **MEME** | NA | 1 (aa64) *** |  |  |
| **iFEL** | 146*** | 0*** |  |  |
| **REL** | 286 | 0 |  |  |

* p-value = 0.1; ** posterior probability = 0.9; *** p-value ≤ 0.05.

*Table S2. Sites under positive and negative selection, for the French PUUV strain dataset (N protein, 433 aa). SLAC: single-likelihood ancestor counting; FEL: fixed-effects likelihood; MEME: mixed effects model of evolution; FUBAR: fast unbiased approximate Bayesian; aBSREL: adaptive branch-site random effects likelihood; BUSTED: branch-site unrestricted statistical test for episodic diversification.*
